# Inhibitory hippocampus-medial septum projection controls locomotion and exploratory behavior

**DOI:** 10.3389/fnsyn.2023.1042858

**Published:** 2023-04-06

**Authors:** Yuh-Tarng Chen, Rachel Arano, Jun Guo, Uzair Saleem, Ying Li, Wei Xu

**Affiliations:** Department of Neuroscience, The University of Texas Southwestern Medical Center, Dallas, TX, United States

**Keywords:** hippocampus, septum, GABAergic interneuron, inhibitory synapse, locomotion, exploratory behavior

## Abstract

Although the hippocampus is generally considered a cognitive center for spatial representation, learning, and memory, increasing evidence supports its roles in regulating locomotion. However, the neuronal mechanisms of the hippocampal regulation of locomotion and exploratory behavior remain unclear. In this study, we found that the inhibitory hippocampal synaptic projection to the medial septum (MS) bi-directionally controls the locomotor speed of mice. The activation of the MS-projecting interneurons in the hippocampus or the activation of the hippocampus-originated inhibitory synaptic terminals in the MS decreased locomotion and exploratory behavior. On the other hand, the inhibition of the hippocampus-originated inhibitory synaptic terminals in the MS increased locomotion. Unlike the septal projecting interneurons, the activation of the hippocampal interneurons projecting to the retrosplenial cortex did not change animal locomotion. Therefore, this study reveals a specific long-range inhibitory synaptic output from the hippocampus to the medial septum in the regulation of animal locomotion.

## Introduction

Moving and exploring freely in an environment are essential skills for constructing a neuronal representation of the world and hence survival. Locomotion is regulated by brain motor systems consisting of brain regions such as the motor cortex, basal ganglia, thalamus, and spinal cord (Ferreira-Pinto et al., 2018). Normally, the hippocampus is considered not a component of this system but rather a cognitive center that integrates multi-modal sensory information and spatial/temporal relations to construct a representation and memory of the world (Eichenbaum, 2004; Buzsaki and Moser, 2013). However, accumulating evidence indicates a direct role of the hippocampus in regulating locomotion.

First, hippocampal neuronal activities, especially those in the dorsal hippocampus, are associated with locomotion. Most noticeably, the activity level of some hippocampal neurons directly reflects the speed of animals (McNaughton et al., 1983; Geisler et al., 2007; Gois and Tort, 2018; Iwase et al., 2020). Interestingly, many of these “speed cells” are found to be inhibitory GABAergic “interneurons” (Gois and Tort, 2018; Iwase et al., 2020). Second, lesions or pathogenic damages to the hippocampus are frequently accompanied by alterations in locomotion (Sams-Dodd et al., 1997; Katsuta et al., 2003; Godsil et al., 2005; White et al., 2006). Third, direct functional manipulations of the hippocampal neuronal activities with optogenetic or pharmacogenetic stimuli altered animal locomotion (Bender et al., 2015; Wolff et al., 2018). However, many studies reported the opposite results. For example, the electrolytic lesion of the hippocampus caused hyperlocomotion (Douglas and Isaacson, 1964) and enhanced the locomotion-stimulating effect of amphetamine (Swerdlow et al., 2001), while the aspiration lesion of the hippocampus did not produce similar impacts (Douglas and Isaacson, 1964). Furthermore, unlike in the study mentioned earlier, some pharmacological, optogenetic, or pharmacogenetic treatments of the hippocampus failed to change animal locomotion (Zhang et al., 2002; Degoulet et al., 2008; Goshen et al., 2011; Lopez et al., 2016; Bian et al., 2019).

The contradictory results may arise from the anatomical and functional heterogeneity of the hippocampus. The hippocampus has multiple functional divisions along its longitudinal and transversal axis (Soltesz and Losonczy, 2018). It consists of numerous neuronal types including both excitatory glutamatergic principal neurons and inhibitory GABAergic interneurons (Pelkey et al., 2017). It is possible that different divisions or cell types were preferentially impacted in the aforementioned studies and generated different behavioral phenotypes. To test this possibility, we focused on the GABAergic outputs from the hippocampus to examine their specific contributions to the regulation of locomotion. We found that among hippocampal GABAergic outputs, those to the medial septum (MS) were particularly engaged in locomotion regulation. Consistent with earlier studies showing the importance of the hippocampus-to-lateral septum pathway (Bender et al., 2015), this study reveals a new hippocampal mechanism for locomotion regulation.

## Materials and methods

### Animals

8- to 12-week-old C57BL/6J (B6J) male mice were obtained from UT Southwestern animal breeding core or The Jackson Laboratory. We used heterozygotes (±) NDNF-Cre transgenic male mice and maintained them on a C57BL/6J background (The Jackson Laboratory). Animal work was approved and conducted under the oversight of the UT Southwestern Institutional Animal Care and Use Committee and complied with the Guide for the Care and Use of Laboratory Animals by the National Research Council.

### AAV vectors

AAV-Dlx-SynaptoTAG was made by switching the Synapsin promoter to the Dlx promoter in the SynaptoTAG2 AAV vector (Li et al., 2021). We also added the coding sequence of GAP43 palmitoylation sequence “MLCCMRRTKQVEKNDEDQKIE” to the 5′ of the tdTomato coding sequence. Construction of the other AAV vectors, packaging, and tittering of AAVs was the same as previously described (Guo et al., 2021). The titers of the AAVs for stereotaxic intracranial injections were in the range of 1E13 to 3E13 copies/ml.

### Stereotaxic surgery and adeno-associated virus injection

Mice were anesthetized with an intraperitoneal (i.p.) injection of tribromoethanol (Avertin) (125–250 mg/kg) before the stereotaxic surgery or anesthetized with 1–3% isoflurane and placed on a stereotaxic instrument (Kopf Instruments). To identify the septum-projecting hippocampal GABAergic interneurons, AAV-Dlx-SynaptoTAG (0.5 μl) was injected into the CA1 (coordinates A/P −1.95 mm, M/L ±1.25 mm, and D/V 1.25 mm). To manipulate the septum-projecting hippocampal GABAergic interneurons, AAV2-retro-Cre (0.75 μl) was injected into the septum (coordinates: A/P 0.80 mm, M/L ±0.00 mm, and D/V 4.20 mm) and infused slowly over 7.5 min (rate: 0.1 μl/min), and then AAV-dlx-DIO-hM3Dq-mCherry was injected into the CA1 (coordinates A/P −1.95 mm, M/L ± 1.25 mm, and D/V 1.25 mm).

To optogenetically activate the hippocampal inhibitory output to the septum, AAV-Dlx-DIO-ChIEF-EGFP and AAV-Syn-Cre were mixed in the ratio of 4:1 (to optically inhibit the hippocampal inhibitory output to the septum, we used the virus mixture of AAV-Dlx-DIO-Jaws-EGFP and AAV-Syn-Cre). Viruses (0.5 μl for each target) were infused slowly over 5 min (rate: 0.1 μl/min) into the CA1 (coordinates A/P −1.95 mm, M/L ±1.25 mm, and D/V 1.25 mm) bilaterally using a microdriver with a 10 μl Halmiton syringe connected to a glass pipette; after the virus injection, flat-cut 400 μm diameter optic fiber with ferrule (Ø 400 μm; CFM14U-20, Thorlabs) was implanted on the top of the medial septum (A/P 0.60 mm, M/L 0.00 mm, and D/V 2.50 mm) and cemented in place using dental cement and CandB-Metabond (Patterson Dental, MN).

To pharmacogenetically manipulate the hippocampal output to the septum, the viruses, AAV-dlx-DIO-hM3Dq-mCherry and AAV-Syn-Cre, were mixed in the ratio of 4:1 (0.5 μl for each target) using the same stereotaxic coordinates to target the CA1. After the virus injection, a gauge 28 guide-cannula was implanted on the top of the medial septum (A/P 0.60 mm, M/L 0.00 mm, and D/V 2.50 mm) and cemented in place using dental cement and CandB-Metabond (Patterson Dental, MN).

To pharmacogenetically manipulate the NDNF-expressing interneurons in the hippocampus, we bilaterally injected 0.5 μl of AAV hDlx-DIO-hM3Dq-mCherry in the dorsal hippocampus (A/P: −1.95, mm, M/L: +1.25 mm, and D/V: 1.45 mm) of NDNF-Cre+ and Cre− littermates.

### Brain slice electrophysiology

After >8 weeks of injection of AAVs (AAV-Dlx-DIO-ChIEF-EGFP and AAV-Syn-Cre mixed in the ratio of 4:1), coronal slices of the septum (300 μm) were prepared with a vibratome (Leica VT1200) in an ice-cold cutting solution containing (in mM) 75 sucrose, 85 NaCl, 2.5 KCl, 1.3 NaH_2_PO4, 4 MgSO_4_, 0.5 CaCl_2_, 24 NaHCO_3_, and 25 D-glucose, saturated with 95% O_2_/5% CO_2_. The slices were incubated for 30 min in artificial cerebrospinal fluid (ACSF) containing (in mM) 124 NaCl, 5 KCl, 1.2 NaH2PO4, 26 NaHCO3, 10 D-glucose, 1.3 MgCl2, and 2.5 CaCl2 at 32°C and then incubated for at least 1 h at room temperature. The cutting solution and ACSF were adjusted to a pH of 7.3–7.4, 290–300 mOsm, and constantly aerated with 95% O_2_/5% CO_2_. The whole-cell patch-clamp recording was performed in a recording chamber perfused (~1 ml/min) with oxygenated ACSF at 26–28°C. The recording pipettes (2.2–3 MΩ) were filled with an internal solution containing (in mM) 120 CsCl, 5 NaCl, 10 HEPES, 10 EGTA, 1 MgCl2, 3 Mg-ATP, 0.3 GTP, and 10 QX-314, adjusted to a pH of 7.3–7.4 and 310 mOsm. For optogenetic experiments, blue light (473 nm) was delivered by an LED coupled with a 40× water objective. Some of the recordings were conducted with Picrotoxin (50 μM) and CGP55845 (10 μM) in the ACSF.

### Optogenetic and pharmacogenetic manipulation

For optogenetics in the ChIEF experiment, a blue laser was delivered through a fiber optic cord using a DPSS Blue 473 nm laser source (MBL-III-473/1–100 mW, Opto Engine LLC). A train of blue laser pulses (10 mW, 20 Hz, 10 ms duration, and 40 ms interval) was generated and controlled by an Optogenetics Pulser (Prizmatix). In Jaw's experiments, continuous orange-red LED was delivered through a fiber optic cord using a high-power orange-red ~625 nm LED module at 10 mW (Prizmatix). The light intensity was calibrated with the PM100D Console (Thorlabs). For pharmacogenetic experiments, i.p. injections of clozapine (0444, Tocris Bioscience) (0.1 mg/kg) or control vehicle saline (0.9% NaCl) were administered 30 min before the behavioral test. For terminal manipulation experiments, intracranial injections of 300 nl clozapine (0.001 mg/ml) or control vehicle saline (0.9% NaCl) were administered 15 min before the behavioral test.

### Open-field test

Animals were handled for 1–2 min a day for 7 days before the open-field test. The open-field apparatus was a custom-made 50 × 50 cm testing chamber. A video camera was placed above the open field, and mice traces were tracked using the ANY-maze video tracking system. Mice were placed in the center of the open-field area prior to the initiation of tracking. The center of the open field was defined as 20 × 20 cm square in the geometric center of the arena. Each chamber was cleaned between individual animal testing. To calculate the percentage of the open-field area each mouse explored, the open-field arena was divided into 100 grids (5 × 5 cm). If the mouse passed through the corresponding grid, then the grid would be counted as being explored. The exploration percentage of the open field ranges from 1 to 100%.

### Elevated plus maze

Animals used in the elevated plus maze (EPM) were tested in the open field before. The EPM apparatus was elevated 38.7 cm above the floor and consisted of two open arms (30.5 cm in length and 5 cm in width) and two closed arms (30.5 cm in length; 6 cm high wall; and 5 cm in width). Open arms and closed arms were all connected to a center platform in the middle (5 cm in length and width). The behavior was recorded and analyzed by the ANY-maze video tracking system.

### Tissue processing, immunohistochemistry, and cell counting

For regular preparation with no need to do immunohistochemical staining, mice were anesthetized by an intraperitoneal (i.p.) injection of tribromoethanol (Avertin) or anesthetized with 1–3% isoflurane and were perfused with phosphate-buffered saline (PBS) followed by 4% paraformaldehyde (PFA) in PBS. Brains were post-fixed in 4% PFA overnight and were cryoprotected in 30% sucrose. Brains were cut into 40 μm sections on a cryostat (Leica CM1950) and were collected in PBS and stored at 4°C. Finally, sections were then mounted on slides and stained with DAPI. Sections were imaged on a Zeiss LSM 880 confocal microscope with a 5×, 10×, and 20× objective under the control of Zen software.

For c-Fos immunohistochemistry staining, mice were injected with clozapine (0.1 mg/kg) and then transferred to a new clean cage and singly housed for 1 h before the perfusion. Brains were post-fixed in 4% PFA overnight and were cryoprotected in 30% sucrose. Brains were cut into 30 μm sections on a cryostat (Leica CM1950) and were collected in PBS. Sections were washed in PBS and blocked in 10% horse serum, 0.2% bovine serum albumin (BSA), and 0.5% Triton X-100 in PBS for 2 h at room temperature. For immunohistochemistry staining, sections were incubated overnight in primary antibodies [anti-cFos antibody: 1:1,000, catalog # 226 003, Synaptic Systems (SYSY)] with 1% horse serum, 0.2% BSA, and 0.5% Triton X-100 in PBS at 4°C. Sections were washed in PBS and reacted with fluorescent secondary antibodies (goat anti-rabbit Alexa Fluor 488, 1:500, Invitrogen, catalog # A-11034) in 1% horse serum, 0.2% BSA, and 0.5% Triton X-100 in PBS for 2 h at room temperature. Sections were then mounted on slides and stained with DAPI. Sections were imaged on the Zeiss LSM 880 confocal microscope with a 5×, 10×, and 20× objective under the control of Zen software.

To quantify the number of activated hM3Dq expressing septum-projecting inhibitory cells in the hippocampus, the hippocampus was outlined as a region of interest (ROI), and the colocalization ratio was calculated as [(cFos^+^ and mCherry^+^)/(mCherry^+^)] × 100. To quantify the fluorescence intensity of the hippocampal projections in each innervated region, the mean intensity of each ROI was acquired using ZEISS ZEN Microscope Software. The normalized fluorescence intensity was calculated as (mean fluorescence intensity)/(average mean fluorescence intensity of the medial septum).

### Statistical analysis

Data are presented as mean ± SEM, and all statistical analyses of the data were performed using GraphPad Prism software (GraphPad Software Inc., La Jolla, USA). Student's unpaired *t*-test was used to analyze two independent samples and the paired *t*-test was used to analyze two dependent samples. To test the optical stimulation effects in two groups, open-field results were analyzed by two-way repeated measures ANOVA with “Order” and “Light” as within-subject factors followed by multiple comparisons tests. A 1 h (5-min bin) open field was analyzed by two-way repeated measure ANOVA with time (minutes) as a within-subject factor and “Group” as a between-subject factor followed by multiple comparisons tests. The Kolmogorov–Smirnov test was used to compare the speed distribution of the two groups. The Mann–Whitney *U*-test was used to compare c-Fos activity (**Figure 3B**) and speed latency (**Figure 4H**). A *p*-value of <0.05 was considered statistically significant.

## Results

### GABAergic hippocampus-septum projections

**Figure 1:**
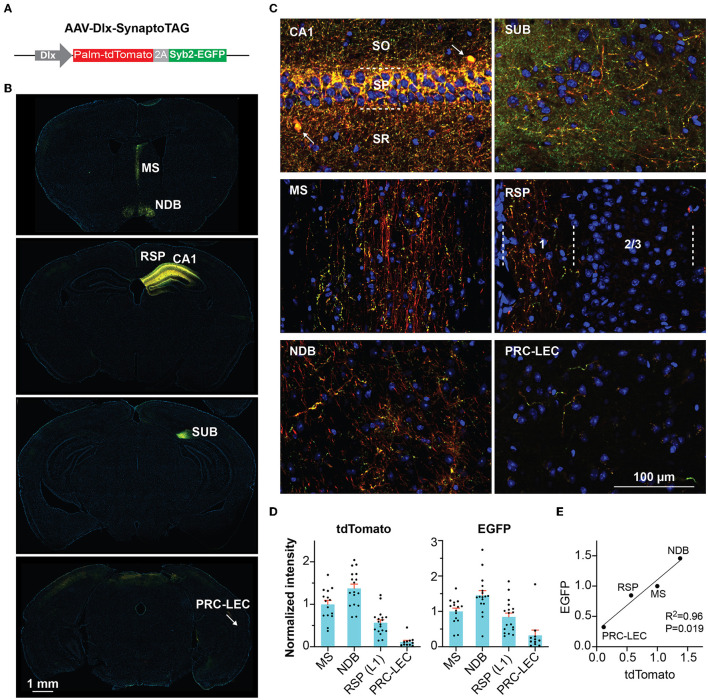
Tracing GABAergic outputs from the hippocampal CA1 region. **(A)** Design of AAV-Dlx-SynaptoTAG. **(B, C)** Low- and high-resolution representative images showing the Palm-tdTomato-positive axons and EGFP-positive boutons in the respective brain regions. The axons and boutons from CA1 GABAergic neurons were detected at the hippocampus, subiculum (SUB), medial septum (MS), and nucleus of the diagonal band (NDB), and the superficial portion of layer 1 of the retrosplenial area (RSP), and the transition area between the perirhinal cortex and the entorhinal cortex (PRC-EC). **(D)** Quantification of the fluorescence intensities in the different brain regions. The intensities were measured from 11 to 17 sections for each region from four mice. Data are represented as mean ± SEM **(E)** Correlation between the averaged intensities of EGFP and tdTomato measured in the brain regions.

Although commonly referred to as “interneurons,” many GABAergic inhibitory neurons in the hippocampus send long-range projections to extrahippocampal regions (Klausberger and Somogyi, 2008). In this study, we focused on the role of these hippocampal inhibitory outputs in regulating locomotion. We first examined the distribution of the long-range hippocampal GABAergic projections. We constructed an adeno-associated virus (AAV) vector (AAV-Dlx-SynaptoTAG) to trace from GABAergic neurons (Figure 1A). This AAV expresses a green fluorescent protein, EGFP, fused with synaptic vesicle protein, Synaptobrevin-2, to label synaptic terminals, and tdTomato to fill the soma and axons, under the control of GABAergic neuron-specific Dlx promoter (Dimidschstein et al., 2016). The tdTomato is fused to the palmitoylation sequence of GAP43 to increase the axonal targeting of the fusion protein (Palm-tdTomato). We injected this AAV into the CA1 region of the dorsal hippocampus. In CA1, we observed the soma of labeled interneurons and densely distributed perisomatic synapses in the pyramidal layer, and less dense synaptic terminals in the other layers (Figures 1B, C). Inside the hippocampal formation (but outside CA1), the subiculum (SUB) had the highest density of EGFP-positive boutons. Outside the hippocampus, tdTomato and EGFP were detected in limited brain regions, including the medial septum (MS), the nucleus of the diagonal band (NDB), the superficial portion of layer 1 of the retrosplenial area (RSP), and to a much less degree, the transition area between the perirhinal cortex (PRC) and entorhinal cortex (EC), consistent with earlier studies (Gulyas et al., 2003; Jinno et al., 2007; Muller and Remy, 2018) (Figures 1B–E).

**Figure 2:**
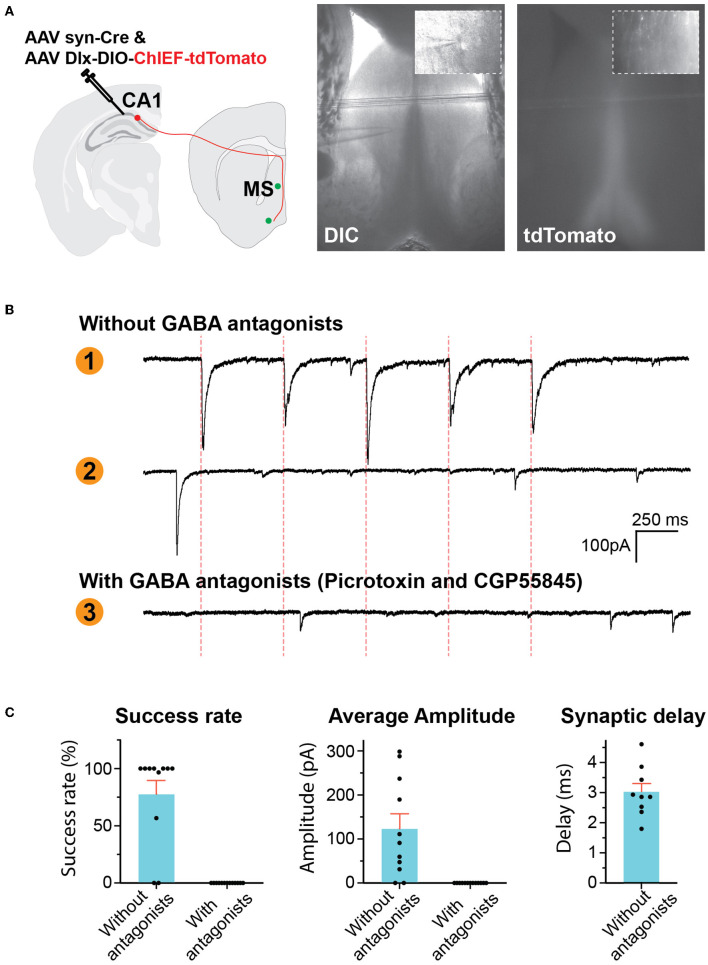
GABAergic hippocampus–septum projections. **(A)** AAV injection scheme (left). Representative photos of brain slice (right) showing the location of tdTomato-positive axons in the MS and the location of whole-cell patch-clamp recording. The inserts are high-resolution images of the location of the pipette tip. **(B)** Representative traces of voltage-clamp recording of postsynaptic currents (PSCs). Traces 1 and 2 were recorded in the absence of GABA antagonists, showing a neuron reacting to optical stimulation (Trace 1) and another neuron not responding (Trace 2). Trace 3 was recorded in the presence of picrotoxin and CGP55845. The red dashed lines indicate the time of optical stimuli. **(C)** Quantification of the PSCs.

To confirm that the traced synaptic boutons at the MS are functional synapses, we expressed excitatory channelrhodopsin ChIEF in the CA1 GABAergic interneurons so that we can activate these neurons by light. We locally injected two AAVs into the CA1: one expressing recombinase Cre and the other ChIEF-tdTomato under the Dlx promoter in a Cre-dependent manner. Cre's expression would turn on the expression of ChIEF-tdTomato. Approximately 2 months after the AAV injection, we prepared acute brain slices containing the MS for whole-cell patch-clamp recordings (Figure 2A). We recorded neurons close to the midline of the septum either in the middle of or right next to the dense axonal bundles running in the midline. Among the 11 neurons from three mice recorded in the absence of antagonists of GABA receptors, optical stimuli elicited postsynaptic currents (PSCs) in nine neurons (Figures 2B, C). A total of eight out of the nine showed success rates (the possibility of each light pulse to evoke PSCs) of 100%, and one neuron had a lower success rate of 56.6%. To determine if the optically evoked PSCs were inhibitory synaptic currents, we recorded neurons in the presence of GABA receptor antagonists, including the GABA-A receptor blocker picrotoxin and the GABA-B receptor antagonist CGP55845. With these antagonists, optical stimuli did not elicit PSCs in the 13 neurons we recorded from two mice although some spontaneous synaptic activities (presumably excitatory postsynaptic currents) could still be observed, indicating that the optically elicited PSCs were GABAergic.

### Activation of septum-projecting hippocampal interneurons decreases locomotion

**Figure 3:**
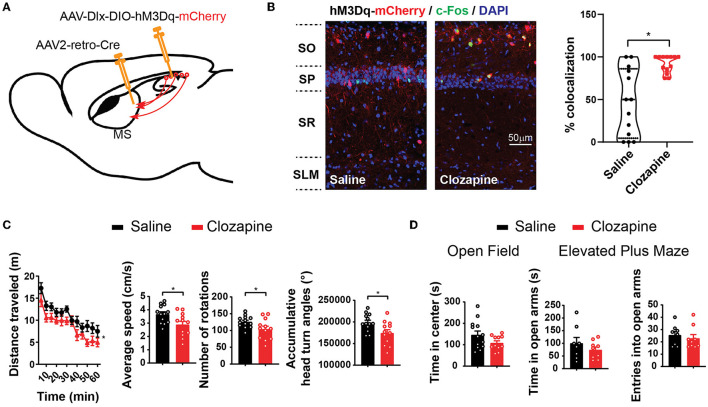
Activation of septum-projecting interneurons reduces locomotion. **(A)** Expression of hM3Dq in septum-projecting hippocampal GABAergic interneurons by AAV injections. **(B)** Intraperitoneal injections of clozapine (0.1 mg/kg), a ligand of hM3Dq, increased activity of septum-projecting hippocampal GABAergic interneurons measured by double labeling of c-Fos (green) and mCherry (red) (saline, *n* = 13 sections, total 62 cells from two mice; clozapine, *n* = 13 sections, total 73 cells from two mice) (Mann–Whitney *U*-test, *p < 0.05). **(C)** Activation of the septum-projecting hippocampal GABAergic interneurons with clozapine decreased mouse locomotion measured by the distance traveled in the open field, number of rotations, and accumulative head turn angles [two-way ANOVA, *F*_(1,23)_ = 6.35, *p* < 0.05; Two-tailed *t*-test, *p < 0.05]. **(D)** Activation of the septum-projecting hippocampal GABAergic interneurons did not change time spent in the center of the open-field test (two-tailed *t*-test, *p* = 0.1), time spent in the open arms (two-tailed *t*-test, *p* = 0.31), and the number of entries into the open arms of the elevated plus maze test (two-tailed *t*-test, *p* = 0.55) (open field: saline: *n* = 13; clozapine: *n* = 11. Elevated plus maze: saline: *n* = 8; clozapine: *n* = 9).

To functionally control these septum-projecting hippocampal GABAergic interneurons, we injected AAV2-retro-Cre into the MS and injected Cre-dependent AAV (AAV-Dlx-DIO-hM3Dq-mCherry) into CA1 (Figure 3A). AAV2-retro-Cre can enter the synaptic terminals that lie in the MS but originate from the hippocampal neurons and is transported back to the soma in CA1 (Tervo et al., 2016). At CA1, the expression of Cre turns on the expression of hM3Dq that only occurs in interneurons due to the Dlx promoter. hM3Dq is an excitatory Designer Receptors Exclusively Activated by Designer Drugs (DREADD) effector that can be activated by its ligand clozapine or clozapine N-oxide (CNO) (Roth, 2016; Gomez et al., 2017). With immunostaining of c-Fos, an immediate early gene reporting neuronal activities, we confirmed that a low dose of clozapine (0.1 mg/kg, intraperitoneal injection) increased the activity of hM3Dq-positive hippocampal GABAergic interneurons, which project to the septum (Figure 3B). We then examined the mice in behavioral tests. In an open field, the mice decreased the speed of locomotion and exploratory behavior with the injection of clozapine (Figure 3C; Supplementary Figure 1) without any change in the time spent in the center of the open-field arena, a parameter frequently used to monitor the anxiety level (Figure 3D). Similarly, the mice did not show a phenotype in an elevated-plus maze test, another frequently used assay for anxiety (Figure 3D). In addition to anxiety, the septum is known to be involved in the expression of other emotional or motivational behaviors. We did not observe other apparent behavioral abnormalities in these mice after the treatments. These results indicate that septum-projecting hippocampal GABAergic interneurons negatively regulate mouse locomotion.

### Activation of hippocampal inhibitory synapses in the MS decreases locomotion

**Figure 4:**
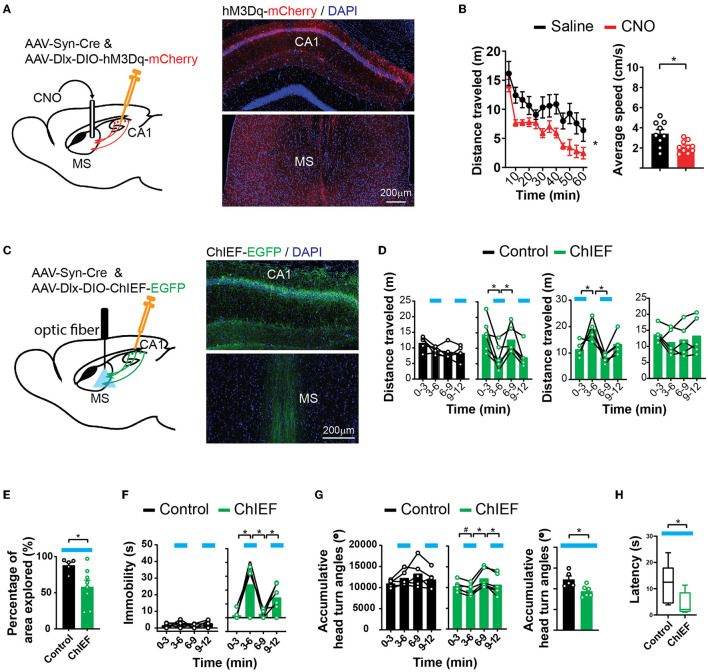
Activation of hippocampal inhibitory projections to the MS reduces locomotion. **(A)** Pharmacogenetic activation of the hippocampal inhibitory inputs to the MS with local infusion of CNO in the MS. **(B)** CNO decreased locomotion in the open field as measured by distance traveled [two-way ANOVA, *F*_(1,17)_ = 9.00, *p < 0.05] and average speed (two-tailed *t*-test, *p < 0.05) (saline, *n* = 9 mice; CNO, *n* = 10 mice). **(C)** Optogenetic activation of the hippocampal inhibitory inputs to the MS by light. An excitatory channelrhodopsin, ChIEF, was expressed in the hippocampal GABAergic neurons with AAVs in the “ChIEF” mice. Control mice received AAVs expressing EGFP only. **(D–H)** Light delivered to the MS (indicated by blue bars) decreased mouse locomotion measured by distance traveled **(D)**, percentage of the open-field area the mice explored **(E)**, immobility **(F)**, accumulative angles of head turns **(G)**, and latency to reach a low speed **(H)**. [**(D)** Two-way ANOVA. Left ChIEF group, light, *F*_(1,5)_ = 30.42, *p* < 0.05; Right ChIEF group, light, *F*_(1,4)_ = 10.15, *p* < 0.05; Tukey's multiple comparison test, *p < 0.05. **(E)** Two-tailed *t*-test, *p < 0.05. **(F)** Left two-way ANOVA. Control group, light, *F*_(1,4)_ = 5.409, *p* = 0.08; ChIEF group, light, *F*_(1,5)_ = 10.57, *p* < 0.05; Tukey's multiple comparison test, *p < 0.05. **(G)** Left two-way ANOVA. Control group, Light, *F*_(1,4)_ = 0.03, *p* = 0.86; ChIEF group, light, *F*_(1,5)_ = 22.01, *p* <0.05; Tukey's multiple comparison test, *p < 0.05; Right two-tailed *t*-test, *p < 0.05. **(H)** Mann–Whitney *U*-test, *p < 0.05.] (Control, *n* = 5 mice; ChIEF, *n* = 6 mice).

The septum-projecting hippocampal GABAergic interneurons may regulate locomotion by inhibiting the local hippocampal network or through their actions in the MS. To determine if their synapses in the MS are sufficient to regulate locomotion, we stimulated these synaptic terminals with locally injected CNO through cannula implanted into the MS to activate hM3Dq (Figure 4A). The injection of CNO into the MS decreased locomotion (Figure 4B), similar to the effects produced by activating the septum-projecting hippocampal GABAergic interneurons (Figure 3C), suggesting that through the direct inhibition of axons in the MS, the septum-projecting hippocampal GABAergic interneurons negatively regulate locomotion.

To temporally precisely control the projections to the MS, we expressed ChIEF in hippocampal interneurons and delivered light to the MS to stimulate the GABAergic hippocampal axons there (Figure 4C). Optical stimulation acutely decreased mouse locomotor activities measured by multiple parameters including the distance traveled (Figure 4D), the area of the open field that the animals explored (Figure 4E), immobility time (Figure 4F), and accumulative head turn angles in the open field (Figure 4G). The stimulation also decreased the latency to reach a low speed (Figure 4H). The decreased locomotion was reversible (Figure 4D) and was not accompanied by a change in the anxiety level (Supplementary Figure 2). Consistent with the aforementioned pharmacogenetic findings, these results indicate that the activation of the GABAergic septal projections from the hippocampus decreases animal locomotion and exploratory behavior.

### Inhibiting hippocampo-septal inhibitory synaptic projections increases locomotion

**Figure 5:**
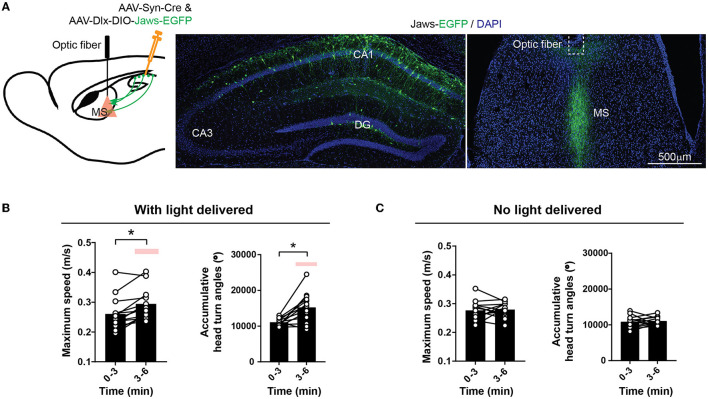
Inhibiting inhibitory hippocampal output to the MS increases locomotion. **(A)** Optogenetic inhibition of the hippocampal inhibitory inputs to the MS by light delivered into the MS. An inhibitory opsin, Jaws, was expressed in the hippocampal GABAergic neurons with AAVs. **(B)** Red light delivered to the MS increased mouse locomotion measured by maximum speed and accumulative head turn angles in the open field. (Two-tailed paired *t*-test, *p < 0.05) (Dlx-Jaws, *n* = 13 mice). The red bars indicate the delivery of optostimulation. **(C)** No change in maximum speed (left) or accumulative head turn angles (right) occurred when the optical stimulation was not delivered (Left: Two-tailed paired *t*-test, *p* = 0.77; Right: Two-tailed paired *t*-test, *p* = 0.71) (Dlx-Jaws, *n* = 13 mice).

Next, to test if the inhibitory hippocampal outputs to the MS could bi-directionally regulate locomotion, we expressed an inhibitory opsin, Jaws (Chuong et al., 2014), in the hippocampal interneurons and delivered light to the MS to silence the GABAergic axons originating from the hippocampus (Figure 5A). This optical inhibition increased mouse locomotor activity measured by the maximum locomotor speed, accumulative head turn angles (Figures 5B, C), and the number of rearing and habituation (Supplementary Figure 3). Nevertheless, the increased locomotion and exploratory behavior were not accompanied by changes in the anxiety level measured by the time spent in the center of the open field or time spent in the open arms of the elevated plus maze (Supplementary Figure 3). Together, the results demonstrate that the activities of the GABAergic septum projection from the hippocampus bi-directionally regulate mouse locomotion and exploratory behavior.

### Retrosplenial cortex-projecting hippocampal interneurons does not change locomotion

**Figure 6:**
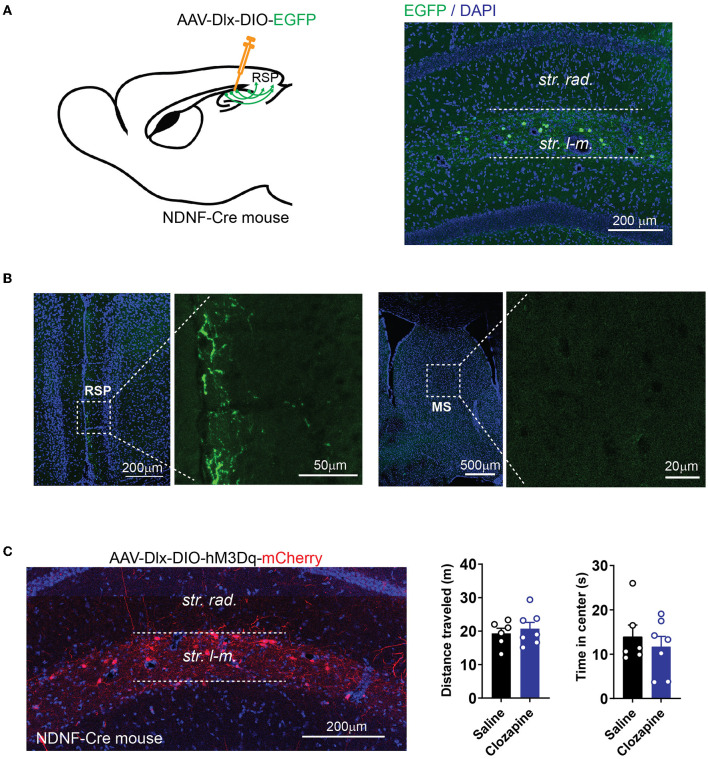
Activation of cortex-projecting hippocampal interneurons does not change locomotor activity. **(A)** Expression of EGFP in NDNF-positive interneurons in NDNF-Cre mice by injecting AAV-Dlx-DIO-EGFP into the hippocampus. **(B)** NDNF-positive interneurons in the hippocampus project to the RSP but not MS. **(C)** Expression of hM3Dq in NDNF-positive interneurons in the hippocampus (left). Pharmacogenetic activation of NDNF-positive interneurons with clozapine did not alter the distance traveled or time spent in the center of the open field (distance traveled: two-tailed *t*-test, *p* = 0.58; time in the center: two-tailed *t*-test, *p* = 0.53; data are represented as mean ± SEM; *n* = 6 mice for the saline group; *n* = 7 mice for the clozapine group).

In addition to the MS, the RSP receives a significant amount of GABAergic inputs from the hippocampus (Klausberger and Somogyi, 2008; Witter, 2010) (Figure 1). Recently, we found that a group of GABAergic interneurons at stratum lacunosum-moleculare (SLM) of the hippocampus, which expresses a marker gene, neuron-derived neurotrophic factor (NDNF), sends long-ranged projection exclusively to the RSP (Figures 6A, B). To determine if the RSP-projecting hippocampal interneurons also regulate locomotion, we expressed hM3Dq selectively in these NDNF-expressing interneurons by injecting the Cre-dependent AAV into the hippocampus of the NDNF-Cre mouse line. Although pharmacogenetic stimulation of these NDNF-cells by an i.p. injection of clozapine significantly changed learning and memory behaviors (Guo et al., 2021), this treatment did not alter mouse locomotion (Figure 6C). It is possible that not enough interneurons were activated in this experiment to significantly alter locomotion. Nevertheless, these results demonstrate the functional heterogeneity of the hippocampal interneurons in the regulation of locomotion and that hippocampal interneurons' impact on locomotion can be separated from their regulation of other behaviors to a certain degree.

## Discussion

In brain systems, the hippocampus lies at the top of the hierarchy of sensory information processing (Squire et al., 2004). The associative cortices feed multi-modal sensory information to the hippocampus to construct an integrated representation and record of the world that includes the “where, when, and what” information about our experiences (Eichenbaum, 2004). The information about an animal's own locations and actions is essential for constructing and updating this neuronal representation (Gois and Tort, 2018). It is therefore not surprising that hippocampal neuronal activities closely correlate with locomotion. However, it was not clear if and how the hippocampus directly regulates locomotion by interacting with brain motor systems and what the functional importance of this regulation may be.

Knowing how the hippocampus directly regulates locomotion will help us to understand not only the neuronal control of locomotion but also the pathogenesis and treatment of neuropsychiatric disorders involving functional abnormalities in the hippocampal circuits. One prominent example is schizophrenia. Schizophrenia patients consistently show anatomical, biochemical, and functional alterations in the hippocampus (Tamminga et al., 2010). In addition to cognitive symptoms, the patients frequently show motor symptoms of either dyskinesia or parkinsonism (Walther and Strik, 2012) and deficits in sensory-motor gating (Braff and Geyer, 1990; Mena et al., 2016). In animal models of schizophrenia, including both pharmacological and genetic models, hyperlocomotion is the most common behavioral phenotype and is commonly considered as a correlate of the positive symptoms (van den Buuse, 2010). The reduction of hyperlocomotion is frequently used as a behavioral readout for screening antipsychotic medicines (Powell and Miyakawa, 2006; Peleg-Raibstein et al., 2008; van den Buuse, 2010; Wolff et al., 2018). Determining the hippocampal mechanisms in locomotion regulation will elucidate the origin of the locomotor symptoms in patients and help us to determine the predictive value of the locomotor parameters in animal models for therapeutic treatments of patients.

In this study, we identified the inhibitory hipocampo-septal projection as a key pathway for locomotion regulation. The pharmacogenetic and optogenetic techniques allowed us to isolate this pathway for precise and reversible functional manipulations. The results indicate that the GABAergic hippocampo-septal projection bi-directionally and reversibly regulates locomotor activities. This reveals a new function of the extrahippocampal projection of the hippocampal interneurons and builds a foundation for further elucidation of hippocampal regulation of motor activities. The MS is innervated by and projects to multiple brain regions. It is engaged in learning, memory, emotional reaction, defensive behaviors, and sensorimotor gating (Tsanov, 2017, 2018; Jin et al., 2019). However, anatomically and functionally, it is most closely coupled with the hippocampus (Muller and Remy, 2018; Iyer and Tole, 2020). The hippocampo-septal loop is particularly critical for generating hippocampal oscillations arising from locomotion and active exploration in the environment (O'keefe and Nadel, 1978; Buzsaki, 2002; Drieu and Zugaro, 2019). MS also plays a pacemaker role in hippocampal theta oscillation (Buzsaki, 2002; Tsanov, 2017). These GABAergic projections to the septum may exert their locomotion regulation effects by altering the functions of the hippocampo-septal loop or acting on the motor structures downstream of the septum.

MS contains GABAergic, cholinergic, and glutamatergic neurons (Huh et al., 2010; Takeuchi et al., 2021). The cholinergic and glutamatergic neurons are mainly in the lateral zones of the MS, while GABAergic neurons are predominantly in the midline zone. Our tracing of the GABAergic axons from the hippocampus shows that these axons and their synapses are largely confined to the midline of MS where GABAergic neurons dominate. An earlier study, which characterized the inhibitory projections from the hippocampus to the MS with optogenetics, showed that ~24% of MS neurons, mainly fast-spiking cells, showed fast IPSCs in response to the optostimulation of hippocampal outputs, while another ~19% of MS cells, such as cholinergic cells, showed synaptic responses to the optical stimulation (Mattis et al., 2014). In the current study, a higher percentage (nine out of 11 recorded cells) of neurons reacted to the optogenetic stimulation of the hippocampal projection with fast IPSCs. This discrepancy may be explained by the differences in the two experimental conditions. In the earlier study, AAV with the somatostatin (SST) promoter was used to express opsins in the rat hippocampus; we used the Dlx enhancer/promoter, which may allow us to express opsins in broader GABAergic neuronal types in the hippocampus. In addition, we conducted the recordings in the neurons close to the axonal bundles at the midline, where neurons responsive to hippocampal projections are enriched. Considering that GABAergic neurons are the predominant cell type in the MS (especially in the midline region) and that >80% of cells we recorded responded to the optical stimulation of the hippocampal projections, we can speculate that the majority of the neurons in the MS innervated by the hippocampal GABAergic neurons are also GABAergic. It is known that the GABAergic neurons in the MS project to the hippocampus and form synapses on the GABAergic interneurons there (Freund and Antal, 1988). The GABAergic neurons in the MS and those in the hippocampus may therefore form a bi-neuronal dis-inhibitory loop. This loop may serve as a positive-feedback mechanism for the GABAergic neurons in the MS to enhance their own activities. The activity of the GABAergic septum-to-hippocampus projection correlates with locomotion (Kaifosh et al., 2013). Consistent with this finding, we saw a decrease in locomotion with the activation of the hippocampus-to-septum GABAergic projections and vice versa. We can speculate that in a behaving animal, the excitatory inputs into the hippocampus may generate an oscillatory activation of the GABAergic interneuron in the hippocampus, which in turn acts on the MS GABAergic neurons to promote or reduce locomotion and exploratory behaviors.

Multiple types of GABAergic neurons exist in the MS, including those expressing calbindin (CaBP), calretinin (CR), or parvalbumin (PV). The PV neurons are more concentrated in the midline and are the projection neurons. These PV neurons are most likely the postsynaptic neurons to the hippocampal GABAergic projections due to their location. The CaBP and CR neurons are local interneurons interacting with the cholinergic and glutamatergic neurons in the MS (Ang et al., 2017). The PV neurons and cholinergic send broad efferent projections to innervate brain regions including the hippocampus, cortex, thalamus, hypothalamus, and brain stem structures. Many of these brain regions, such as the motor cortex, the lateral habenula in the thalamus, and the lateral hypothalamus, have been shown to interact with the septum in regulating locomotion (Bender et al., 2015; Zhang et al., 2018). Therefore, determining the cell types in the MS receiving hippocampal inhibitory innervation and their synaptic projections to other locomotion-related brain regions will be essential for us to draw a complete picture of how the hippocampo-septum system regulates locomotion and how this regulation is coupled to the cognitive functions of this circuit. Recently, we developed techniques for stepwise polysynaptic tracing and genetic control (Du et al., 2021; Li et al., 2021). These techniques allow us to trace and selectively control the septal neurons innervated by the hippocampal GABAergic outputs and their postsynaptic neurons in other brain regions. With these techniques, we may be able to identify the next stage of the hippocampo-septal pathway in regulating locomotion.

## Data availability statement

The original contributions presented in the study are included in the article/Supplementary material, further inquiries can be directed to the corresponding author.

## Ethics statement

The animal study was reviewed and approved by UT Southwestern Institutional Animal Care and Use Committee.

## Author contributions

Y-TC and WX designed this study. Y-TC, RA, JG, US, YL, and WX conducted the experiments, collected data, and plotted the figures. Y-TC wrote the first draft of this paper. All authors contributed to the writing and approved the submitted version.
