# Supplementary material for: Inhibitory hippocampus-medial septum projection controls locomotion and exploratory behavior
Source: Front Synaptic Neurosci. 2023 Apr 6;15:1042858. doi: 10.3389/fnsyn.2023.1042858 (PMC10116069; doi:10.3389/fnsyn.2023.1042858)
Supplement: Supplementary file 1 [file Data_Sheet_1.PDF]

Inhibitory Hippocampus-medial septum Projection
Controls Locomotion and Exploratory Behavior

Supplemental information

Supplemental Figure 1

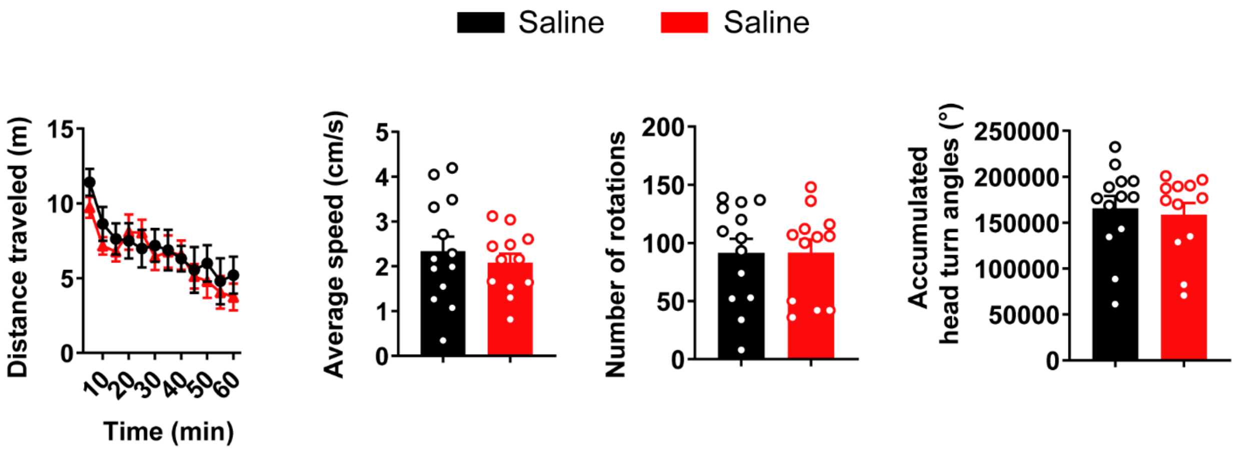

Supplemental Figure 1. Injection of saline produced no impacts on locomotion (distance traveled: Two-way ANOVA, $F(1,23)=0.24$, $p=0.63$; number of rotations: Two-tailed t-test, $p=0.99$; accumulated head turn angles: Two-tailed t-test, $p=0.71$).

Supplemental Figure 2.

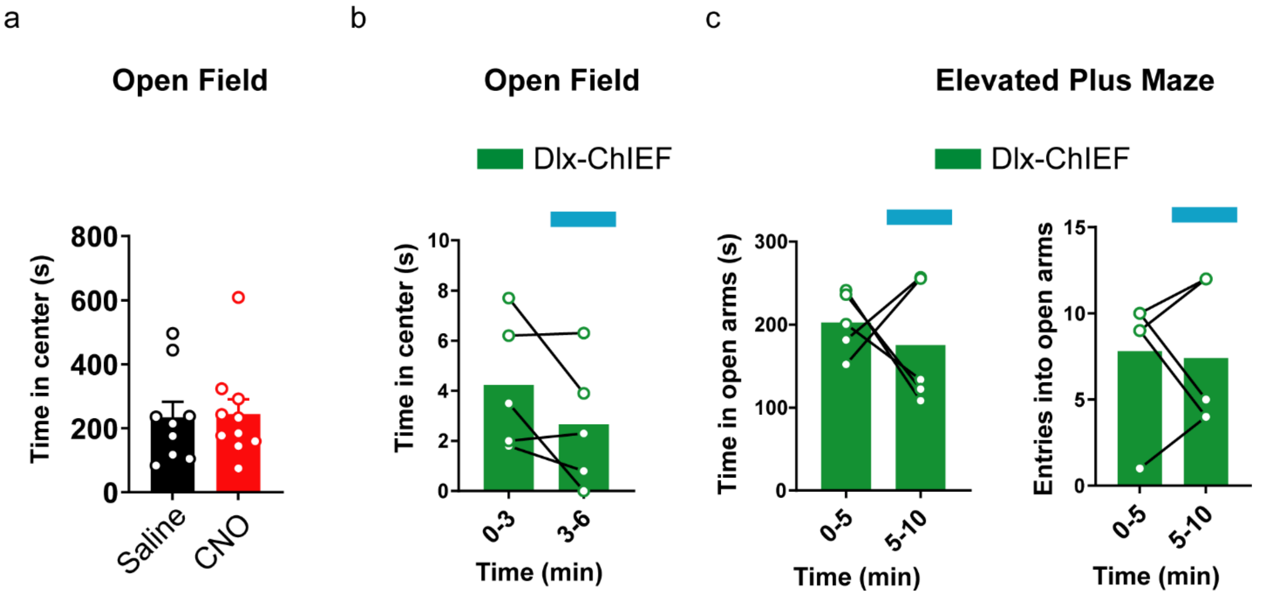

Supplemental Figure 2. Activation of hippocampal inhibitory synaptic inputs to the MS did not change anxiety level. (**a**) Pharmacogenetic activation of the hippocampal inhibitory inputs to the MS did not change animals time spent in the center of the open field (Two-tailed t-test, p=0.89) (GroupA, n=9 mice; GroupB, n=10 mice). (**b**) Optogenetic activation of the hippocampal inhibitory inputs to the MS did not change time spent in the center of the open field (Two-tailed paired t-test, p=0.15) (n=5 mice). (**c**) Optogentic activation of the hippocampal inhibitory inputs to the MS did not change time spent in the open arms (Two-tailed paired t-test, p=0.61,) or the number of entries into the open arms of the elevated plus maze (Two-tailed paired t-test, p=0.84) (n=5 mice).

Supplemental Figure 3

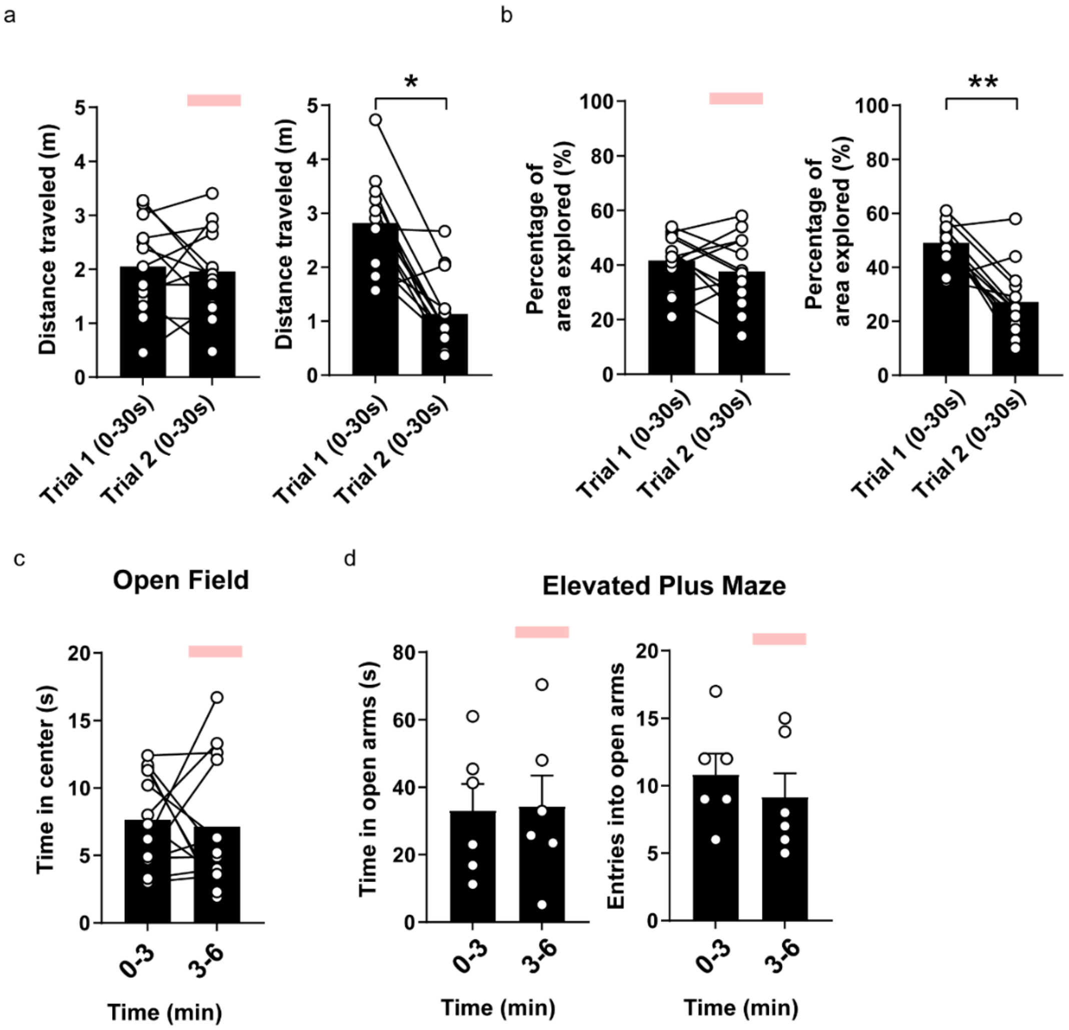

Supplemental Figure 3. Optogenetic inhibition of the hippocampal inhibitory inputs to the MS disrupts locomotion habituation but does not change anxiety level. (**a**) Optogenetic inhibition of the hippocampal inhibitory inputs to the MS disrupted locomotion habituation (Two-tailed paired t-test, p=0.72, *p<0.05). (**b**) Optogenetic inhibition of the hippocampal inhibitory inputs to the MS maintained the percentage of the open field area animals explored (Two-tailed paired t-test, p=0.29, *p<0.05). (**c**) Optogenetic inhibition of the hippocampal inhibitory inputs to the MS did not change the animals time spent in the center of the open field (Two-tailed paired t-test, p=0.77). (**d**) Optogenetic inhibition of the hippocampal inhibitory inputs to the MS did not change the animals time spent in the open arms (Two-tailed paired t-test, p=0.87) and number of entries into the open arms of the elevated plus maze (Two-tailed paired t-test, p=0.48) (Dlx-Jaws, n=13 mice).
